# Using poly(N-Vinylcaprolactam) to Improve the Enzymatic Hydrolysis Efficiency of Phenylsulfonic Acid-Pretreated Bamboo

**DOI:** 10.3389/fbioe.2021.804456

**Published:** 2021-11-30

**Authors:** Xianqing Lv, Guangxu Yang, Zhenggang Gong, Xin Cheng, Li Shuai, Liulian Huang, Lihui Chen, Xiaolin Luo, Jing Liu

**Affiliations:** ^1^ College of Materials Engineering, Fujian Agriculture and Forestry University, Fuzhou, China; ^2^ Jiangsu Provincial Key Laboratory of Pulp and Paper Science and Technology, Nanjing Forestry University, Nanjing, China

**Keywords:** lignin, non-productive adsorption, enzymatic hydrolysis, poly(N-vinylcaprolactam), phenylsulfonic acid

## Abstract

Chemical pretreatment followed by enzymatic hydrolysis has been regarded as a viable way to produce fermentable sugars. Phenylsulfonic acid (PSA) pretreatment could efficiently fractionate the non-cellulosic components (hemicelluloses and lignin) from bamboo and result in increased cellulose accessibility that was 10 times that of untreated bamboo. However, deposited lignin could trigger non-productive adsorption to enzymes, which therefore significantly decreased the enzymatic hydrolysis efficiency of PSA-pretreated bamboo substrates. Herein, poly(N-vinylcaprolactam) (PNVCL), a non-ionic surfactant, was developed as a novel additive for overcoming the non-productive adsorption of lignin during enzymatic hydrolysis. PNVCL was found to be not only more effective than those of commonly used lignosulfonate and polyvinyl alcohol for overcoming the negative effect of lignin, but also comparable to the robust Tween 20 and bovine serum albumin additives. A PNVCL loading at 1.2 g/L during enzymatic hydrolysis of PSA pretreated bamboo substrate could achieve an 80% cellulosic enzymatic conversion and meanwhile reduce the cellulase loading by three times as compared to that without additive. Mechanistic investigations indicated that PNVCL could block lignin residues through hydrophobic interactions and the resultant PNVCL coating resisted the adsorption of cellulase via electrostatic repulsion and/or hydration. This practical method can improve the lignocellulosic enzymatic hydrolysis efficiency and thereby increase the productivity and profitability of biorefinery.

## Introduction

Transforming renewable carbohydrates into platform chemicals (such as ethanol and lactic acid) via biochemical conversion (enzymatic hydrolysis and fermentation) methods has become an effective way to reduce the dependence of human society on petrochemical products (Ragauskas et al., 2006). To avoid the competition with humans and poultry for raw materials, the research focus has gradually shifted from edible carbohydrates (e.g., corn starch and sucrose) to inedible lignocelluloses (Shuai et al., 2016a; Luo et al., 2020b; Luo et al., 2022). However, the structurally dense linkages between three macromolecular polymers (i.e., cellulose, hemicellulose and lignin) in lignocelluloses makes the release of sugars from carbohydrates difficult and costly (Himmel et al., 2007).

Compared to five-carbon sugars (e.g. xylose), six-carbon sugars (e.g. glucose) are more easily metabolized by microorganisms into secondary metabolites such as ethanol or lactic acid (Ho Nancy et al., 1998). As such, cellulose that solely polymerized from glucose, has been regarded as an ideal substrate for producing fermentable six-carbon glucose through enzymatic hydrolysis (Pan et al., 2005; Sun and Cheng, 2005). To improve enzymatic accessibility of cellulose to enzymes, different thermochemical pretreatments (e.g., dilute acid, alkaline, organosolv, deep-eutectic solvents and hydrotropic acid) have commonly used to remove non-cellulosic components (e.g., hemicellulose, lignin, or both of them) from lignocelluloes prior to enzymatic hydrolysis (Zhu and Pan, 2010; Shuai et al., 2016b; Liu et al., 2019). Significant removal of non-cellulosic components indeed enables the deconstruction of lignocellulose and therefore improves the accessibility of cellulose in pretreated substrates to enzymes (Yang and Wyman, 2004). However, the accessibility mentioned above can only be termed as “potential accessibility” since it will not necessarily resulted in high cellulose conversion (Liu et al., 2017; Shi et al., 2018). In many cases, high enzyme loadings are still needed because hydrophobic lignin re-distributed or re-deposited on the surface of pretreated substrate would adsorb some of the enzymes (Selig et al., 2007; Donohoe et al., 2008). As lignin has a higher adsorption capacity for cellulase than cellulose (Ko et al., 2015), deposited lignin can competitively adsorbs enzyme molecules with cellulose, resulting in less enzyme molecules that can be used for cellulose hydrolysis (Luo et al., 2019; Luo et al., 2020a; Gong et al., 2021). As a result, more enzymes should be loaded for achieving considerable cellulose conversion, thereby increasing the processing cost of enzymatic hydrolysis.

In addition to chemical or genetic modification of enzymes and lignin (Chen and Dixon, 2007; Brogan et al., 2018), the use of additives for overcoming the lignin’s negative effects during enzymatic hydrolysis has been considered as one of the most practical methods (Yang and Wyman, 2006; Li and Zheng, 2017). The reported additives mainly include non-catalytic proteins, metal ions and surfactants (Liu et al., 2016; Saini et al., 2016; Li and Zheng, 2017). Non-catalytic proteins (such as bovine serum protein, casein, soybean protein and peanut protein) can restrain the non-productive adsorption of enzymes via blocking lignin through hydrophobic and/or hydrogen-bonding interactions (Yang and Wyman, 2006; Florencio et al., 2016; Luo et al., 2019). Unfortunately, as a medical material or an edible nutrient, the cost of non-catalytic proteins is still very high (Klein-Marcuschamer et al., 2012). Metal ions are relatively inexpensive, but the accumulated cations will affect the stability of the buffer system and the activity of microbial cells in downstream fermentation (Tejirian and Xu, 2010). Ionic surfactants such as lignosulfonates and cetyltrimethyl ammonium bromide also have inherent defects similar to those of metal ions (Soodsma and Nordlie, 1969; Lan et al., 2020; Zheng et al., 2020). Therefore, researchers have done intensive studies on development of inexpensive and biocompatible non-ionic surfactants (Eriksson et al., 2002). Many non-ionic surfactants such as Tween 80, Tween 20, Triton X-100, polyethylene glycol (PEG) and polyvinyl alcohol (PVA), had been used to improve the enzymatic hydrolysis efficiency of lignocelluloses (Eriksson et al., 2002; Börjesson et al., 2007; Liu et al., 2016; Saini et al., 2016; Li and Zheng, 2017). However, as non-ionic additives containing similar key structure (-CH_2_CH_2_O-), Triton X-100 inhibited the metabolism of glucose to ethanol by microorganisms, while Tween 80 did not exhibit adverse effects (Lee et al., 1996). This indicates that the inhibition mechanism of non-ionic additives to microorganisms is really complex and should be deeply explored in biological research field. Therefore, in addition to revealing the inhibition mechanism towards microorganisms, further development of novel non-ionic surfactants with different structure will be not only benefit to the enzymatic hydrolysis, but also enrich the types of additives compatible to the subsequent fermentation process.

As an additive of enzymatic hydrolysis, it needs not only to block lignin, but also to resist protein adsorption effectively. Developed non-ionic surfactants (e.g., PEG and Tween 20, etc.) commonly possess (–CH_2_CH_2_O–) unit (Eriksson et al., 2002; Börjesson et al., 2007). The foregoing studies (Kristensen et al., 2007; Li and Zheng, 2017) have speculated that effective adsorption could be well formed between carbon skeleton of non-ionic surfactants and lignin through hydrophobic interaction. Residual ether linkages (–O–) tend to hydrate with water molecules, thereby resisting the adsorption of enzymes on the surface of adsorbed non-ionic surfactants. From the perspective of molecular structure, poly(N-vinylcaprolactam) (PNVCL) contains hydrophobic polyethylene chain and hydrophilic amide bonds. Also, PNVCL has been developed as a drug carrier, indicating its biocompatibility to biological system (Vihola et al., 2002). Inspired by the interaction mechanism of developed non-ionic surfactants and lignin and the amphiphilic polymers [e.g. poly(methacrylic acid)] that used for anti-protein fouling (Wang et al., 2020), PNVCL was therefore explored as an additive for improving the enzymatic hydrolysis efficiency of PSA pretreated bamboo substrate in this study. From the perspective of the interactions between lignin, enzyme and additive, the promoting mechanism of PNVCL was also explored, which could be useful for developing advanced additives and improving the economics of lignocellulosic bioconversion process.

## Materials and Methods

### Materials

Moso bamboo chips that provided by Fujian Hibos Chemical Technology Co., Ltd. (Nanping City, China) were air-dried and carefully milled to pass through 40 mesh for subsequent pretreatment. Chemicals including phenylsulfonic acid (PSA), PVA and Tween 20, and dyes (Congo red and Rose Bengal) were purchased from Aladdin^®^ Chemicals (Shanghai, China). PNVCL with molecular weight ranged from 1.3 to 354 kDa and bovine serum albumin (BSA) were obtained from Shanghai Yuanye Bio-Technology Co., Ltd. (Shanghai City, China) and VWR™ (Shanghai City, China), respectively. Cellulase (Celluclast1.5 L^®^), β-glucosidase (Novozyme 188), microcrystalline cellulose (Avicel^®^ PH-101) and lignosulfonate were ordered from Sigma-Aldrich Company (Shanghai, China). Silicon wafers (boron doping, crystal orientation 100, 5 × 5 mm) used for contact angle measurements were provided by Kaihua Lijing Electronics Co., Ltd. (Zhejiang, China).

### Phenylsulfonic Acid Pretreatment and Lignin Isolation

Bamboo particles (< 40 mesh) were pretreated by PSA aqueous solution in a thick-walled glass bottle. Prior to the pretreatment, an 80 wt. % PSA aqueous solution was prepared at 60°C. The prepared PSA aqueous solution was pre-mixed with bamboo particles in the thick-walled glass bottle, while the mass ratio of PSA solution to bamboo particles (o.d.) was kept at 15:1 (w/w). The thick-walled glass bottle was sealed by a Teflon cap and transferred into the oil bath. The mixture in the thick-walled glass bottle was then magnetically stirred at 500 rpm and heated at 95°C for 30 min. Finally, the pretreatment was stopped by cooling the bottle to room temperature with tap water. The slurry was immediately filtered under vacuum to separate the pretreated solid substrate and pretreatment liquor. The separated solid substrate was washed with de-ionic water until obtaining a neutral filtrate. Actually, it is difficult to completely separate PSA from the pretreated solid substrate during scale-up process because of controlling processing cost. Recently, phenol-4-sulfonic acid, a derivative of PSA, was well recovered by using ethanol to washing pretreated solid substrate, which resulted in a high recovery yield (∼98.3%) of phenol-4-sulfonic acid and would be a potentially effective way to recover PSA (He et al., 2020). Otherwise, neutralization of residual PSA by weak alkali (e.g. Na_2_CO_3_) would also be an alternative method to alleviate its negative effects towards enzymes and microorganism. The dissolved lignin was precipitated by diluting pretreatment liquor with 20 times volume of de-ionic water. The precipitated lignin (termed as PSA lignin) was washed with de-ionic water to neutral, vacuum dried and collected for following experiments.

### Composition Analysis

After air-dried, the compositions of untreated and PSA pretreated bamboo particles were analyzed by a well-developed method (Sluiter et al., 2008). Based on measured substrate yield (SY) and specific component content, the component (cellulose, hemicellulose or lignin) removal can be calculated as followings:

$$RM=100\frac{\left(W_{0}-0.01W_{1}\times SY\right)}{W_{0}}$$ (1)

where RM is the removal (%) of certain component (cellulose, hemicellulose or lignin) after pretreatment; W_0_ and W_1_ refer to the mass content (%) of certain component (cellulose, hemicellulose or lignin) in o. d. raw material and pretreated solid substrate, respectively; SY (%) is the mass yield of solid substrate after pretreatment.

### Characterizations of Untreated Bamboo and Pretreated Substrate

Prior to characterization, untreated bamboo particle and pretreated substrate were further vacuum dried for 12 h at 40°C. The dried samples were sputter-coated with gold and imaged by a scanning electron microscopy (SEM, SU8010, Hitachi, Japan). For evaluating the surface diffuse reflectance property, the dried samples without coating gold were further characterized by a UV-Vis-NIR Systems (Agilent Cary 7000, CA, US) at 200–800 nm with a spectral bandwidth of 2 nm. For differentiating the reflection characteristics of the samples especially in UV region (200–350 nm), the measured reflectivity (R_λ_) was further converted to its F(R) value based on a well-known Kubelka-Munk function (Klaas et al., 1997).

$$F\left(R\right)=\frac{{\left(1-R_{\lambda }/100\right)}^{2}}{2\left(R_{\lambda }/100\right)}$$ (2)

where R_λ_ is the reflectance (%) of sample measured at certain wavelength (nm).

The accessibilities of untreated bamboo particle and pretreated substrate were evaluated by a Congo red staining method (Yu et al., 2020). In general, Congo red dye was pre-dissolved in acetate buffer solution (60 mmol/L and pH 6.0). The solid sample (untreated bamboo particle or pretreated substrate) without additional drying were mixed with a 2 g/L of Congo red buffer solution at a 1% (w/v) concentration in an Erlenmeyer flask. The flask was sealed with a rubber stopper and shaken in an incubator at 50°C and 180 rpm for 24 h. After staining, the solid-liquid mixture was immediately cooled with tap water to room temperature and centrifuged at 740 g for 10 min. The supernatant was filled into a Quartz cuvette and measured by a UV-Vis spectrometer (Agilent 8454, CA, US) at 498 nm. A linear relationship (Absorbance = 19.98 × Congo red concentration + 0.02, *R* ^2^ = 0.99) was developed between Congo red concentration (0.005–0.1 g/L) and absorbance (0.1–2.0) of UV-Vis spectrometer at 498 nm. The accessibility of sample was referred to the amount of adsorbed Congo red dye (mg/g) that could be calculated based on a pre-established standard curve (dye concentration versus absorbance value).

Similarly, the hydrophobicities of these two samples were measured by Rose Bengal staining method (He et al., 2018). A 40 mg/L of Rose Bengal solution was prepared by dissolving dye in citrate buffer solution (50 mmol/L and pH 4.8). The solid sample without further drying was also directly suspended in Rose Bengal buffer solution with a concentration ranges of 2–10 g/L. The incubation was conducted at 50°C and 150 rpm for 2 h in a shaker. At the end of staining, the concentration of dye in separated supernatant was measured by a UV-Vis spectrometer (Agilent 8454, CA, US) at 543 nm. First, the concentration of Rose Bengal (2.5–25.0 g/L) was linearly plotted against measured absorbance (0.1–1.6) of UV-Vis spectrometer at 543 nm. The residual dye concentration after adsorption could be calculated based on initial dye concentration, measured absorbance and developed linear relationship (Absorbance = 0.06 × Rose Bengal concentration−0.02, *R* ^2^ = 0.99). Another linear relationship was then developed between partition quotients (the mass ratio of adsorbed dye to free dye) and loaded sample concentrations (g/ml). The slope (ml/g) of developed linear curve was thus used to denote the hydrophobicity of sample.

### Enzymatic Hydrolysis

The enzymatic hydrolysis of pretreated substrate, microcrystalline cellulose (Avicel) and untreated bamboo were conducted at 50°C and 200 rpm for 72 h in an acetate buffer solution (50 mmol/L). The pH value of buffer solution was adjusted from 4 to 6, while the solid loading in the buffer solution was kept at 2% (w/v). Specifically, about 0.1 g (o.d.) of substrate (PSA-Ba substrate or Avicel) and 5 ml of hydrolysis liquor were performed in a 10-ml centrifuge tube for all hydrolysis experiments. Cellulase and β-glucosidase loadings were 5–20 FPU/g glucan and 7.5–30 CBU/g glucan, respectively. The β-glucosidase loading with a unit of CBU/g glucan was 1.5 times cellulase loading (FPU/g glucan). The concentration of glucose released in the buffer solution was measured by a sugar analyzer (2900D, YSI Inc., Yellow Springs, OH, United States).

To investigate the lignin effect on the enzymatic hydrolysis, PSA-pretreated substrate was soaked in 0.1 mol/L of NaOH or HCl solution at room temperature and 2% (w/v) loading for 2 h and then washed with de-ionic water to neutral before enzymatic hydrolysis. Furthermore, the lignin isolated from the PSA pretreatment liquor was also added into the enzymatic hydrolysis medium of the pretreated substrate or Avicel. The mass ratio of lignin to pretreated substrate or Avicel ranged from 0:1 to 1:1. For overcoming the negative effect of lignin, the additive (PNVCL, Tween 20, BSA, PVA or LS) was pre-mixed with the pretreated substrate in an acetate buffer solution (50 mmol/L, pH 5.0) at 50°C and 200 rpm for 2 h. After that, the enzymes (cellulase and β-glucosidase) were added into the hydrolysis medium and further shaken in the incubator for 72 h at 50°C and 200 rpm.

Based on the measured glucose concentration, the enzymatic conversions (ECs) of glucan in the pretreated substrate or Avicel can be calculated by the following equation.

$$\mathrm{EC}=\frac{0.9C_{\mathrm{glucose}}\times \;V_{buffer}\times D}{0.01W_{sample\;\times \;\gamma }}$$ (3)

where EC refers to the enzymatic conversion (%) of glucan in sample; C_glucose_ and V_buffer_ are the measured glucose concentration (g/ml) of sample and volume (ml) of acetate buffer solution used for the enzymatic hydrolysis; D is the dilution factor for the sample diluted with the fresh acetate buffer solution for glucose concentration measurement; W_sample_ and γ are the o. d. weight (g) of the sample used for enzymatic hydrolysis and corresponding glucan content (%), respectively.

### Quartz Crystal Microbalance Measurement

The adsorption properties of cellulase and PNVCL on the PSA lignin film were evaluated by the quartz crystal microbalance (QCM) (Biolin Corp., Gothenburg, Sweden) (Gong et al., 2021; Zheng et al., 2021). Initially, a PSA lignin film was prepared on the surface of the QCM gold sensor according to a reported spin-coating method (Cai et al., 2017b; Zheng et al., 2021). Sample solution was prepared by dissolving 0.5 g of cellulase or PNVCL in 1 L of acetate buffer solution (50 mmol/L and pH 5.0). After the QCM flow module was thoroughly cleaned by the Milli-Q water, the acetate buffer solution (50 mmol/L and pH 5.0) was imported into the QCM flow module and used to balance the baseline at 23°C and 0.15 ml/min. After that, the sample solution (cellulase or PNVCL, 0.5 g/L) was injected into the QCM flow module at 0.15 ml/min and the corresponding frequency changes (∆*f*) were immediately monitored. When the adsorption reached an equilibrium, the injection of sample solution was stopped and the fresh acetate buffer solution was re-imported into the flow module for evaluating the desorption properties of cellulase or PNVCL. For PNVCL test, the fresh cellulase buffer solution was further injected into the flow module after the desorption reaching an equilibrium. The third overtone of collected data was used to evaluate the frequency changes (∆*f*) of the samples. The adsorption capacity of PSA lignin to cellulase or PNVCL was finally estimated by the Sauerbrey equation (Sauerbrey, 1959; Lai et al., 2019).

$$AC=\frac{-\varphi \times \Delta f}{n}$$ (4)

where AC (ng/cm^2^) presents the adsorption capacity of PSA lignin towards enzyme or additive; *φ* is the QCM constant with a value of 17.7 (ng/cm^2^/Hz); n is the number of overtone and equals to 3.

### Zeta Potential and Contact Angle Analysis

Commercial cellulase or PNVCL was initially dissolved in an acetate buffer solution (50 mmol/L and pH 5.0) for zeta potential analysis. The concentration of the prepared cellulase protein or PNVCL buffer solution was kept at 200 mg/L. To measure the zeta potentials of samples at different pH values, the pH values of the mother acetate buffer solution was adjusted to 3–6 by 1 mol/L of HCl or NaOH aqueous solution. Finally, the zeta potential of the prepared sample was measured by a Malvern Zetasizer (Nano ZS90, Malvern Instruments, Malvern, United Kingdom).

For contact angle measurements, PSA lignin or PNVCL was dissolved in pyridine-acetic acid-water mixture (9:1:4, v/v/v) at a concentration of 2 wt%. The prepared solution was coated on a silicon wafer (boron doping, crystal orientation 100, 5 × 5 mm) via a spin-coating method at 400 rpm for 30 s, 800 rpm for 60 s and 400 rpm for 30 s. The resulted coatings were exposed in HCl steam (37 wt. %) for 30 s and then preserved in de-ionic water. Prior to measurement, the coatings were taken out of the water and thoroughly dried by N_2_. The contact angles of dried coatings were finally measured with pure water on a contact angle tester (DSA30S, Krűss GmbH, Germany), when the water droplets stably existed on the silicon wafers.

## Results and Discussion

### Effects of Lignin on the Enzymatic Hydrolysis

The aforementioned studies (Chen et al., 2017; Luo et al., 2020a) reported that the aryl sulfonic acid-based hydrotropic medium (e.g. *p*-toluenesulfonic acid) could effectively fractionate lignocelluloses. To facilitate the discussion, untreated bamboo and PSA-pretreated bamboo are abbreviated as Un-Ba and PSA-Ba. After the pretreatment, more than 88% of non-cellulosic components (hemicelluloses and lignin) were selectively removed, resulting in a substrate with high glucan content (>84%, Table 1). Theoretically, the substantial removal of non-cellulosic components would be beneficial to improve the accessibility of cellulose in pretreated substrate to enzymes.

**TABLE 1:** The contents and removal of components in untreated bamboo and PSA pretreated solid substrate.

**Samples**	**Temp. (°C)**	**Time (min)**	**PSA loading (%)**	**SY (%)^a^**	**Components content (%)^a^**			**Components removal (%)^b^**		
					**Glucan**	**Hemi^c^**	**K. Lignin^c^**	**Glucan**	**Hemi**	**K. Lignin^c^**
Un-Ba	—	—	—	—	39.7 ± 1.2	24.0 ± 1.3	26.4 ± 1.5	—	—	—
SA-Ba	95	30	80	43.4 ± 1.6	84.3 ± 0.6	6.0 ± 1.1	6.2 ± 0.7	9.8 ± 2.7	88.9 ± 1.6	90.2 ± 1.5

a SY, and Components content were calculated based on the o. d. weight of Un-Ba and PSA-Ba substrate.

b Components removal was calculated based on the o. d. weight of special component in Un-Ba.

c Hemi. and K. Lignin refer to the abbreviations of hemicellulose and Klason lignin, respectively.

**FIGURE 1:**
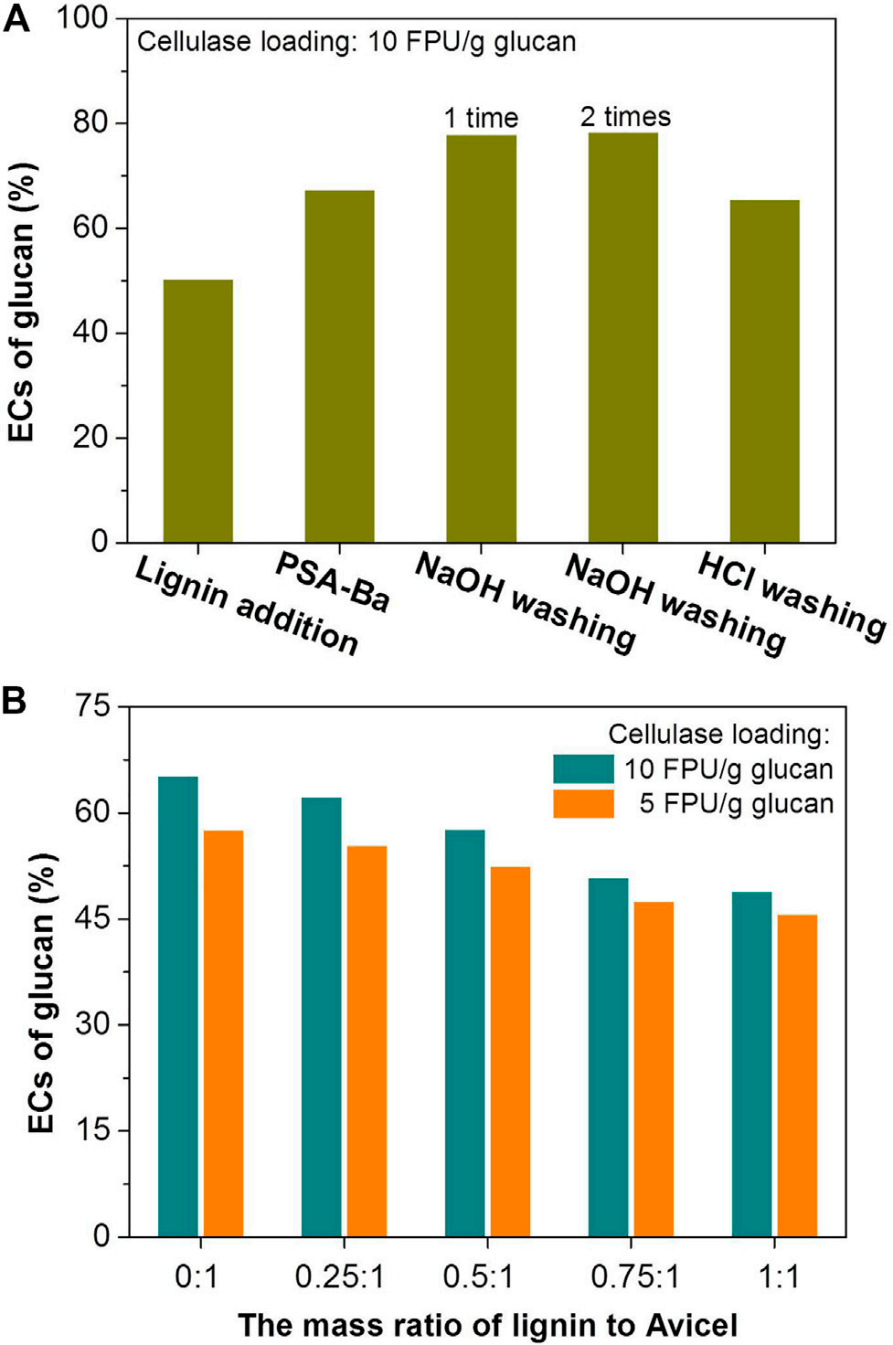
Effects of PSA lignin addition and washing treatment on the ECs of glucan in **(A)** PSA-Ba substrate and **(B)** pure cellulose (Avicel). The mass ratio of added PSA lignin to PSA-Ba substrate (o.d.) shown in Figure 1A was 1:1. The β-glucosidase loadings are 7.5 and 15 CBU/g glucan for the enzymatic hydrolysis with cellulase loadings of 5 and 10 FPU/g glucan, respectively. The pH value of acetate buffer solution is 5.

Although PSA pretreatment can improve the enzymatic conversion (EC) of glucan in the pretreated substrate, high enzymes loadings are still necessary to afford high cellulose conversion. For example, a cellulosic conversion of 67% was achieved for PSA-Ba substrate (Figure 1A), while the EC of glucan in Un-Ba was only 5%. However, such unobtrusive EC was obtained at high cellulase loading (10 FPU/g glucan). Similar phenomena were also reported by previous reports (Chen et al., 2017; Luo et al., 2020a). For PSA-pretreated alkaline peroxide mechanical pulp (APMP), a cellulase loading of 20 FPU/g glucan only resulted in less than 80% of cellulosic EC (Dong et al., 2021). Although the cellulosic EC of poplar wood could reach 90% after *p*-toluenesulfonic acid pretreatment, a cellulase loading as high as 15 FPU/g glucan was still essential (Chen et al., 2017). Some researchers had attributed the need for high cellulase loadings to the lignin deposits (Luo et al., 2020a; Lan et al., 2020), which could significantly adsorb cellulase and reduce the amount of the enzyme molecules involved in cellulose hydrolysis.

Herein, lignin addition and pre-washing were used to verify the above speculation. Under the same enzymatic hydrolysis conditions, adding PSA lignin indeed decreased the EC of glucan in PSA-Ba substrate from 67 to 50% (Figure 1A). Pre-washing PSA-Ba substrate prior to enzymatic hydrolysis with 0.1 mol/L of HCl solution resulted in unobvious changes of cellulosic EC (67 versus 65%), presumably due to the insolubility of PSA lignin in acidic aqueous solutions. Under the same washing conditions, pre-washing of the substrate with 0.1 mol/L of NaOH solution once could increase the cellulosic EC from 67 to 78% (Figure 1A). However, washing the substrate with NaOH solution twice did not further improve the enzymatic hydrolysis efficiency (Figure 1A). This may be due to that washing once would be enough to remove lignin deposits. Increasing the lignin addition also decreased the EC of pure cellulose (Avicel^®^ PH-101) (Figure 1B), which directly proved the negative effect of lignin to the cellulose EC. Therefore, to avoid the generation of alkaline waste, developing efficient additives will be an effective way to overcome the negative influence of residual lignin.

### Characterizations of Untreated Bamboo and Pretreated Substrate

**FIGURE 2:**
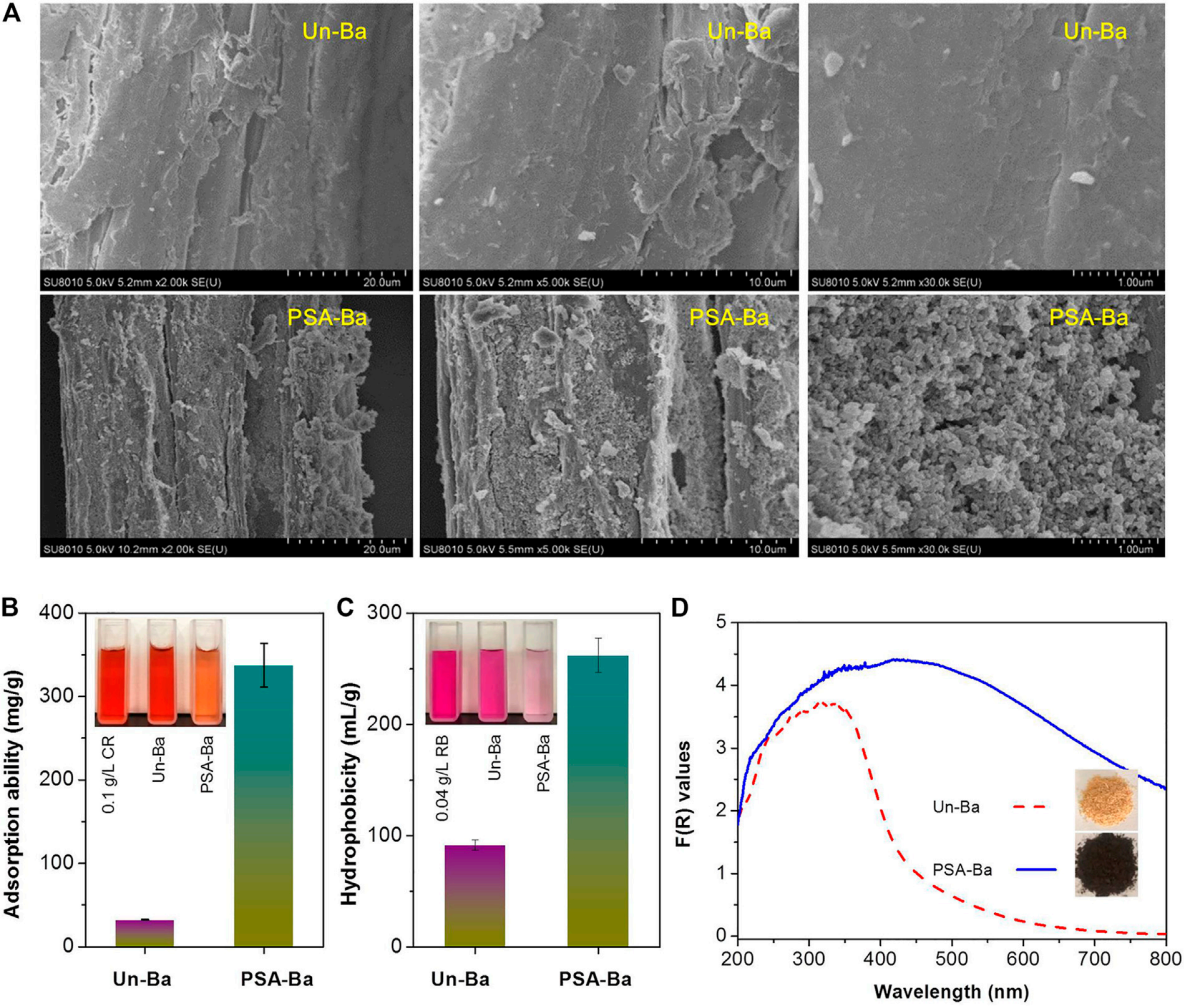
**(A)** SEM images, **(B)** accessibility, **(C)** hydrophobicity and **(D)** F(R) values of Un-Ba and PSA-Ba substrate. F(R) values were calculated based on the measured sample reflectance (%) and Kubelka-Munk function (Eq. 2). CR and RB shown in Figure 2B are the abbreviations of Congo red and Rose Bengal, respectively.

To verify the speculation mentioned above, the Un-Ba and PSA-Ba substrates were characterized by SEM and other methods. SEM results showed that the surface of Un-Ba was relatively smooth (Figure 2A). Without intense mechanical treatments (e.g., stirring or size reduction), the morphology of the PSA-Ba substrate observed by SEM was also relatively complete. However, some small cracks and pores (Figure 2A) still appeared on the surface of the PSA-Ba substrate. This may be caused by the removal of non-cellulosic components (hemicellulose and lignin) (Chen et al., 2017; Dong et al., 2021). Based on the results of the compositional analysis (Table 1), we speculate that the removal of non-cellulose components will result in exposure of cellulose. Due to the specific adsorption of Congo red dye to cellulose, a well-known Congo red staining method (Yu et al., 2020) was used to semi-quantitatively characterize the accessibilities of Un-Ba and PSA-Ba substrates. As expected, the adsorption capacity of PSA-Ba substrate towards Congo red was 10 times higher than that of Un-Ba (Figure 2B), proving the effectiveness of PSA pretreatment for improving substrate accessibility.

However, such considerable substrate accessibility did not yield satisfactory cellulosic EC (<70%) at a relatively high enzyme loading (Figure 1A). These seemingly contradictory results promoted us to further characterize Un-Ba and PSA-Ba substrates. In addition to small cracks and pores, many irregular particles could be also observed on the surface of the PSA-Ba substrate (Figure 2A). This is probably due to the aggregation and re-deposition of dissolved or relocated hydrophobic lignin fragments in aqueous solution, though sulfonic compounds are actually efficient to catalyze the significant cleavage of the aryl ether bonds of lignin (Bian et al., 2017; Chen et al., 2017). In the pretreatments using polar aqueous solutions (such as liquid hot water and organosolv pretreatments), similar phenomena were also revealed previously (Ko et al., 2015; Liu et al., 2017; Shi et al., 2018; Liu et al., 2019; Luo et al., 2019). For this reason, we further characterized the hydrophobicity of these samples. Although PSA pretreatment removed more than 90% of lignin, we still found that the hydrophobicity of PSA-Ba substrate was about 3 times that of Un-Ba (Figure 2C). The F(R) values of the PSA-Ba substrate in UV and Vis regions were also higher than that of Un-Ba (Figure 2D), proving that the former possessed higher absorption capacity towards UV and Vis lights. Since cellulose and hemicellulose do not absorb UV and Vis lights under measurement conditions, this result further supports the fact of that more lignin deposits present on the surface of PSA-Ba substrate as compared to the Un-Ba.

The above-mentioned component analysis (Table 1), NaOH washing (Figure 1), and substrate characterization (Figure 2) comprehensively illustrate that although PSA pretreatment can efficiently fractionate bamboo components, lignin deposits can competitively adsorb enzyme molecules from cellulose, which results in reduced enzymatic hydrolysis efficiency.

### Comparisons of Different Additives for Enzymatic Hydrolysis

In addition to the complex biochemical modifications of enzyme and lignin (Chen and Dixon, 2007; Brogan et al., 2018), the use of additives is a well-tested method for overcoming the adverse effects of lignin deposits (Eriksson et al., 2002; Yang and Wyman, 2006; Börjesson et al., 2007; Kristensen et al., 2007; Wang et al., 2013). In addition to the poly(N-vinylcaprolactam) (PNVCL) that was explored for improving the enzymatic hydrolysis efficiency of PSA solid substrates, the reported representative additives were also used for comparisons.

**FIGURE 3:**
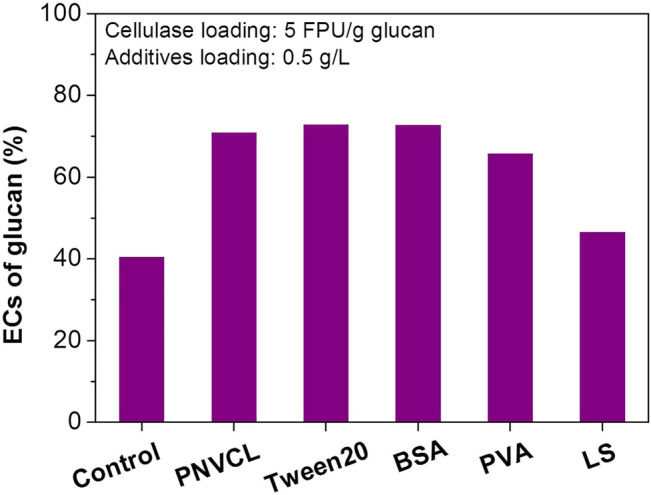
Effects of different additives on the enzymatic hydrolysis efficiencies of PSA-Ba substrate. The β-glucosidase loading is 7.5 CBU/g glucan. PNVCL, BSA, PVA and LS are the abbreviations of poly(N-vinylcaprolactam), bovine serum albumin, polyvinyl alcohol and lignosulfonate, respectively. The molecular weight of PNVCL and pH value of acetate buffer solution used here is the 1.3 kDa and 5, respectively.

At low enzyme loadings, the adverse effect of lignin deposits on the enzymatic hydrolysis becomes more obvious. When the cellulase loading was reduced from 10 to 5 FPU/g glucan, the EC of glucan in the PSA-Ba substrate rapidly decreased from 67% (Figure 1A) to 41% (Figure 3). This is most likely caused by the lignin deposits characterized on the surface of PSA-Ba substrate (Figure 2), which can result in non-productive adsorption to enzymes. The addition of PNVCL at 0.5 g/L may overcome or at least alleviate such negative effect, which increased the glucan EC from 41% for that without additive to 70% at a cellulase loading of five FPU/g glucan (Figure 3). This promotion effect is not only significantly higher than those of PVA and lignosulfonate, but also comparable to Tween 20 and BSA (Figure 3), two of the most robust additives reported so far. Considering the inhibition of Tween 20 to *Saccharomyces cerevisiae* (Peng and Chen, 2011) and the high cost of BSA (Klein-Marcuschamer et al., 2012), PNVCL, a non-ionic surfactant that is structurally different from Tween 20 and PEG, would be therefore used as a novel additive for improving the enzymatic hydrolysis efficiency of PSA-Ba substrate.

### Improvement of Enzymatic Hydrolysis by PNVCL

**FIGURE 4:**
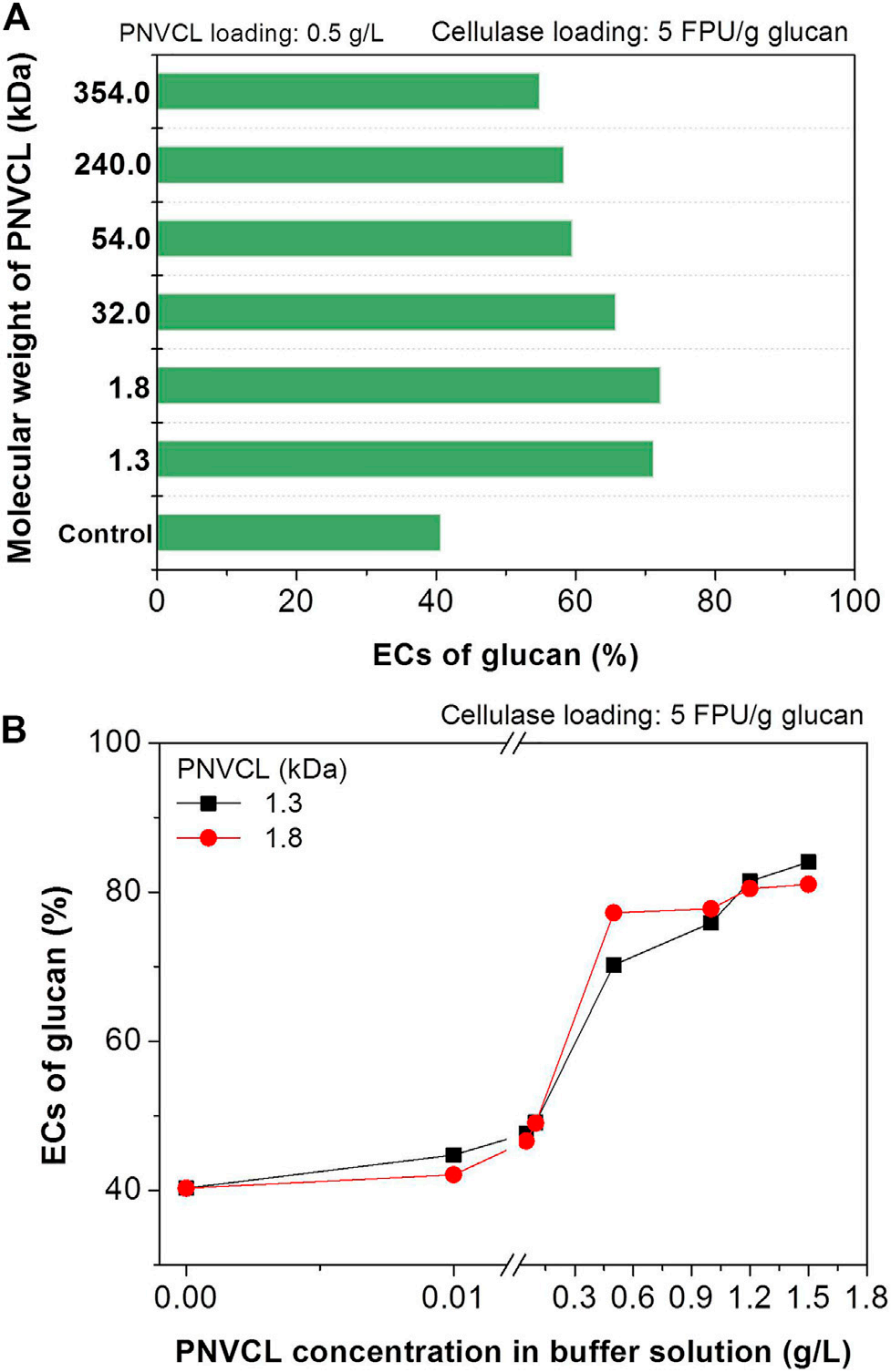
Effects of **(A)** PNVCL molecular weight and **(B)** PNVCL loading on the ECs of glucan in PSA-Ba substrate. The pH value of acetate buffer solution and β-glucosidase loading are five and 7.5 CBU/g glucan. The control experiments shown in Fig.3, Figure 4A and Figure 5 denote the enzymatic hydrolysis of PSA-Ba substrate without additive.

Herein, the factors that affecting the promoting effect of PNVCL to enzymatic hydrolysis will be systematically investigated. With the same enzyme and additive loadings, it can be seen that PNVCL (1.3–354 kDa) of different molecular weights had different promoting effects. For example, after adding PNVCLs of 1.3 and 1.8 kDa molecular weights into the enzymatic hydrolysis medium, the EC of glucan in the PSA-Ba substrate increased from 41% for the control sample to more than 70% (Figure 4A) at a cellulase loading of 5 FPU/g glucan. However, the use of PNVCL of a higher molecular weight (32 kDa) led to a slight decrease in the glucan EC (66%). When the molecular weight of PNVCL increased to more than 300 kDa (e.g. 354 kDa), the corresponding EC further decreased to 55% (Figure 4A). This may be caused by the strengthened inter- and/or intra-molecular aggregations (Maltesh et al., 1992). With polyolefin structure and amide group, increasing molecular weight may facilitate the aggregations of PNVCL through hydrophobic and/or hydrogen-bonding interactions. Therefore, PNVCLs with molecular weights of 1.3 and 1.8 kDa were used in other investigations.

By using low-molecular-weight PNVCLs as additives, the effect of its addition on the enzymatic hydrolysis efficiency was further investigated at a low cellulase loading (5 FPU/g glucan). When the additive loading was lower than 0.1 g/L, the highest EC did not exceed 50%, despite the ECs of glucan in PSA-Ba substrate increased with the increase in PNVCL loading (Figure 4B). After increasing the addition of PNVCL (1.3 and 1.8 kDa) to 0.5 and 1.2 g/L, the corresponding ECs reached 70 and 80%, respectively. However, further increasing the PNVCL loading over 1.2 g/L did not change the enzymatic hydrolysis efficiency of the PSA-Ba substrate (Figure 4B). Therefore, a PNVCL loading of 1.2 g/L is enough to overcome the adverse effects of PSA lignin on cellulase.

**FIGURE 5:**
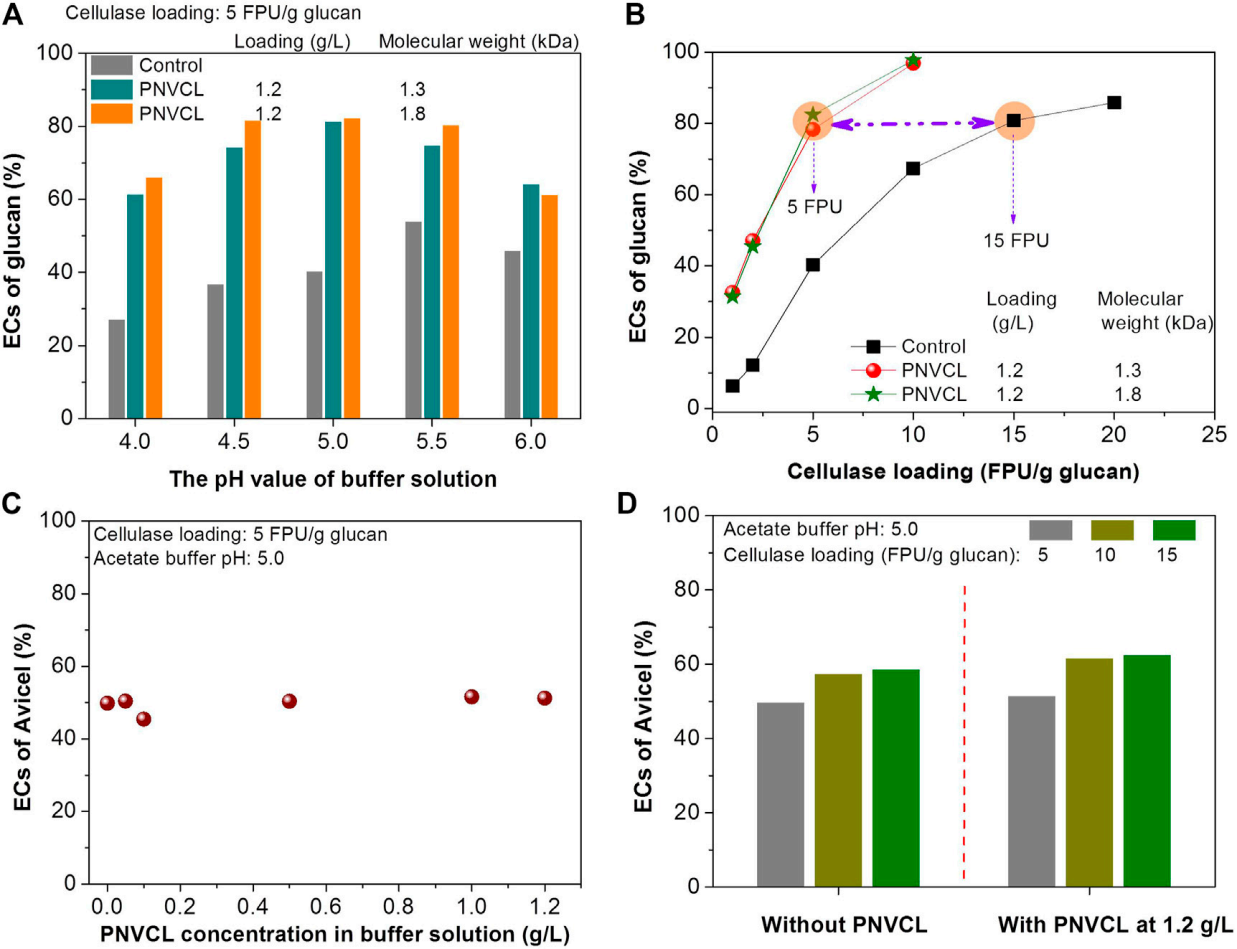
Effects of **(A)** the pH values of acetate buffer solutions and **(B)** cellulase loadings on the ECs of glucan in PSA-Ba substrate; effects of **(C)** and **(D)** PNVCL loading on the ECs of Avicel at different cellulase dosage (5–15 FPU/g glucan). The pH values of acetate buffer solution used in Figure 5B are 5, and the β-glucosidase loading (CUB/g glucan) for each enzymatic hydrolysis is 1.5 times of cellulase loading (FPU/g glucan).

It can be also found that the buffer pH values have different effects on the enzymatic hydrolysis with and without PNVCL. The pH value of the acetate buffer was optimized at 5.5 for the enzymatic hydrolysis of PSA-Ba substrate without additive (Figure 5A). This is mainly due to that the pH value of the acetate buffer solution not only affects the enzyme activity but also changes the surface charges of enzymes and substrate (Lan et al., 2013). The high pH value (e.g. 5.5) can make the surface charge of enzymes and substrate more negative, which can trigger the electrostatic repulsion between the enzymes and substrate and thereby reduce the non-productive adsorption of the enzymes on the substrate (Lan et al., 2013; Lou et al., 2013). For the enzymatic hydrolysis using the low-molecular-weight PNVCLs (1.3 and 1.8 kDa) as additives at 1.2 g/L, the highest glucan EC was obtained in the acetate buffer solution at pH 5 (Figure 5A). After blocking the lignin deposits by PNVCL, we speculate the acetate buffer solution with either too high or low pH value would negatively affect the enzyme activity since the robust activity of commercial cellulase preparation from *Trichoderma reesei* was reported at a narrow pH value range (4.8–5) of acetate buffer solution (Lan et al., 2013). However, the ECs of glucan in the PSA-Ba substrate with PNVCL were significantly higher than those without additives at each investigated pH value (4–6), indirectly indicating that lignin deposits rather than the pH value of the buffer solution induced enzyme activity and surface charge would be the key factor governing the enzymatic hydrolysis efficiency.

In addition to the factors discussed above, the effects of cellulase loadings on the enzymatic hydrolysis efficiencies were further investigated. In order to achieve the same level of glucan EC, the cellulase loading required for enzymatic hydrolysis without additives was significantly higher than that with PNVCL. For example, enzymatic hydrolysis without additive required a cellulase loading of 15 FPU/g glucan to achieve an 80% glucan EC, which was three times (Figure 5B) that with the addition of PNVCL at 1.2 g/L and an acetate buffer pH value of 5. These experimental results again indicate that non-productive adsorption of lignin to enzymes would be the key factor affecting the enzymatic hydrolysis efficiency of PSA-Ba substrate.

Although above results verify the validity of low-molecular-weight PNVCL for improving the enzymatic hydrolysis efficiency, its effect on subsequent fermentation of glucose to produce ethanol or other platform chemicals (e.g. lactic acid) would be the on-going experimental focus. Since the molecular weights of PNVCL (1.3–354 kDa) used in this study are far higher than that of glucose, it can be separated from enzymatic hydrolysate by reported membrane technologies (i.e., *in-situ* membrane bioreactor and off-line ultrafiltration) (Toledano et al., 2010; Al-Zuhair et al., 2013), if it presents no positive effect to glucose fermentation. Otherwise, it can also be separated from fermentation broth using similar membrane technologies after fermentation.

### Promoting Mechanism of PNVCL Towards Enzymatic Hydrolysis

The effectiveness of PNVCL as an enzymatic hydrolysis additive further urged us to explore its promoting mechanism. Previous study (Bhagia et al., 2018) reported that non-ionic surfactants (e.g. Tween 20) could effectively alleviate the inactivation of cellulase at the gas-liquid interface, thus improving the enzymatic hydrolysis efficiency of microcrystalline cellulose (Avicel^®^ PH-101). To validate this effect, enzymatic hydrolysis experiments of Avicel with and without PNVCL were conducted at a volumetric ratio of 10 ml of reactor (centrifuge tube) to ∼5 ml of hydrolysate that used for PSA-Ba substrate. It was found that the ECs of Avicel did not increase with the increase in PNVCL loading (0–1.2 g/L) (Figure 5C) at a cellulase loading of 5 FPU/g cellulose. Moreover, with different cellulase loadings (5, 10, and 15 FPU/g cellulose), there was also no obvious difference in the ECs of Avicel with and without PNVCL (Figure 5D). These results proved that since low volumetric ratio of reactor to hydrolysate (10 vs. 5 ml) used, the role of PNVCL in improving enzymatic hydrolysis efficiency of PSA-Ba substrate would mainly contribute from inhibiting cellulase non-productive adsorption through blocking lignin.

**FIGURE 6:**
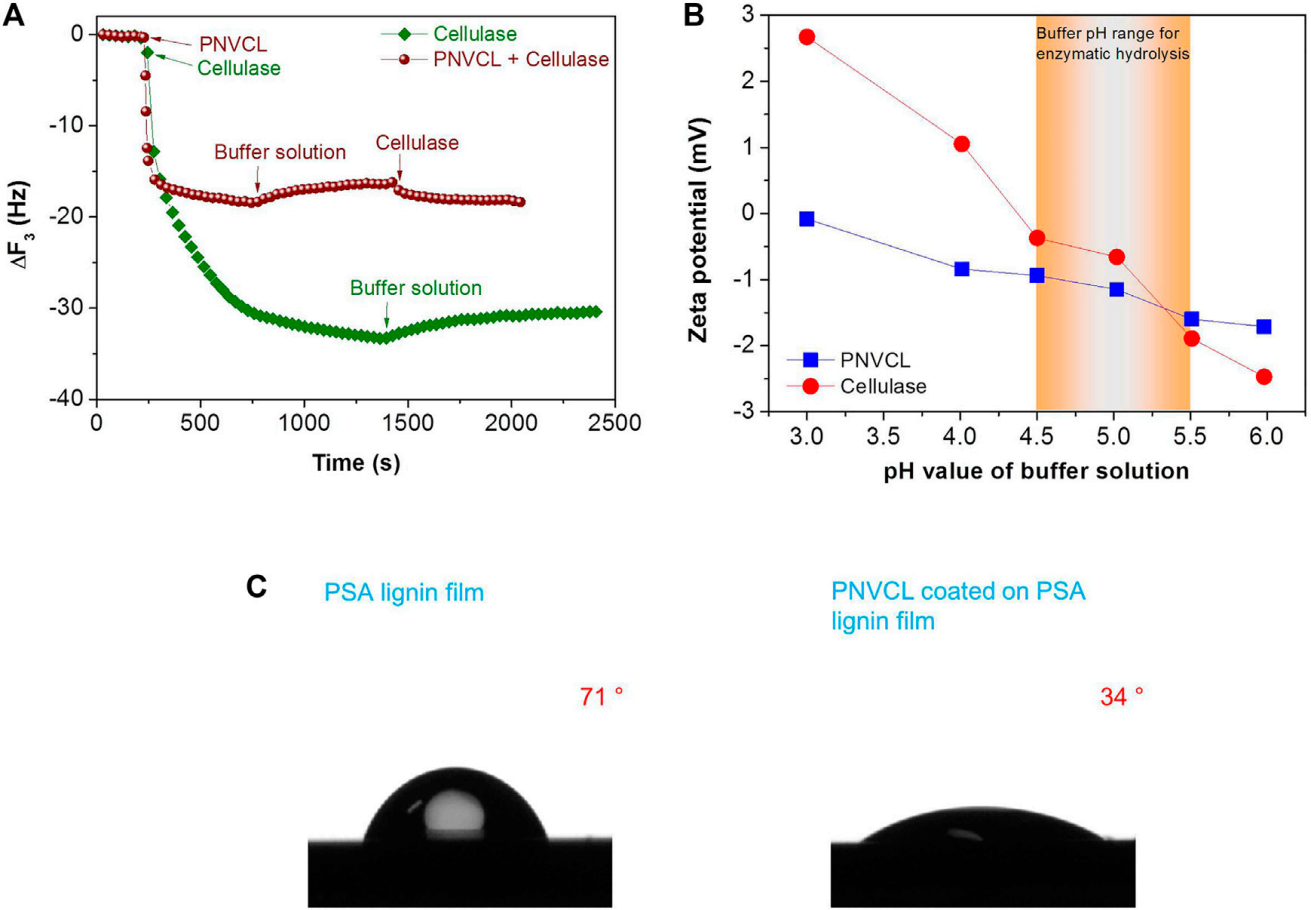
**(A)** The adsorption of PNVCL and cellulase on the PSA lignin film during QCM measurements; **(B)** zeta potentials of PNVCL and cellulase at different pH values; **(C)** contact angles of water on the PSA lignin and PNVCL films. The PNVCL solution was directly coated on the PSA lignin film that was pre-coated on the silicon wafer by a spin-coating method.

To reveal the blocking performance of PNVCL towards lignin, adsorption properties of cellulase and PNVCL on lignin film were investigated by QCM analysis. In the process of QCM analysis, enzymes were quickly absorbed on the PSA lignin film and resulted in a significant frequency changes (*∆f*, 33.3 Hz) (Figure 6A). After fresh buffer solution was injected into the QCM flow module, only 10% of the cellulase (*∆f*, 33.3 to 30.4 Hz) was desorbed, indicating the stable adsorption of cellulases on the PSA lignin film. When the adsorption of PNVCL on PSA lignin film reached equilibrium, introducing fresh acetate buffer solution also resulted in only 2.2 Hz of frequency change (*∆f*, 18.4–16.2 Hz), i.e. only 12% desorption of PNVCL from PSA lignin film. Another 2.1 Hz of frequency change (Figure 6A) was observed for further switching the fresh buffer solution in flow module to the cellulase solution, indicating that only few PSA lignin sites (12%) released by the desorption of PNVCL could be re-adsorbed by cellulase molecules. Overall, these QCM adsorption-desorption results illustrate that PNVCL can not only block lignin firmly, but also effectively restrain cellulase adsorption. As a result, even though the adsorption capacity of PSA lignin towards cellulase (197 ng/cm^2^) was higher than that toward PNVCL (109 ng/cm^2^), PSA lignin-induced non-productive adsorption during enzymatic hydrolysis could be effectively overcome if PNVCL was pre-mixed with the solid substrate in an acetate buffer solution.

In addition to adsorption capacity, the interactions among PNVCL, lignin and cellulase molecules were further analyzed. First, it was found that the surface electronegativity of the PNVCL and cellulase increased with the increase in the pH values of the acetate buffer. The zeta potentials (Figure 6B) were measured as −0.9 to −1.6 for PNVCL and −0.4 to −1.9 for cellulase in the pH range (4.5–5.0) commonly used for enzymatic hydrolysis. In this pH range, the net surface charge of PSA lignin was also reported to be negative (Chen et al., 2017; Luo et al., 2020a). Therefore, we infer that electrostatic attraction (Wang et al., 2013) and cation-π interaction (Zheng et al., 2021) would be not the main factors governing the efficient blocking of PNVCL towards PSA lignin. Because of the negative surface charge, the PNVCL coating formed on the surface of PSA lignin may further inhibit the adsorption of cellulase via electrostatic repulsion.

In addition to electrostatic and cation-π interactions, the hydrophobicities of PSA lignin and PNVCL were further investigated. When PSA lignin was adsorbed on the surface of relatively hydrophobic silicon wafer, the surface of PSA lignin film was still hydrophobic (71°) (Figure 6C). However, the continued spin-coating of PNVCL on the surface of the hydrophobic PSA lignin film contrarily reduced the contact angle of water on the new coating to 34° (Figure 6C). This indicates that PNVCL can not only be adsorbed on the surface of the hydrophobic PSA lignin film, but also form a hydrophilic coating after shielding the PSA lignin. Previous studies have found that hydrophilic surfaces could be easily hydrated, which would facilitate the inhibition of protein fouling induced by the hydrophobic interaction (Cai et al., 2017a; Cai et al., 2017b; Wang et al., 2020; Zheng et al., 2021).

**FIGURE 7:**
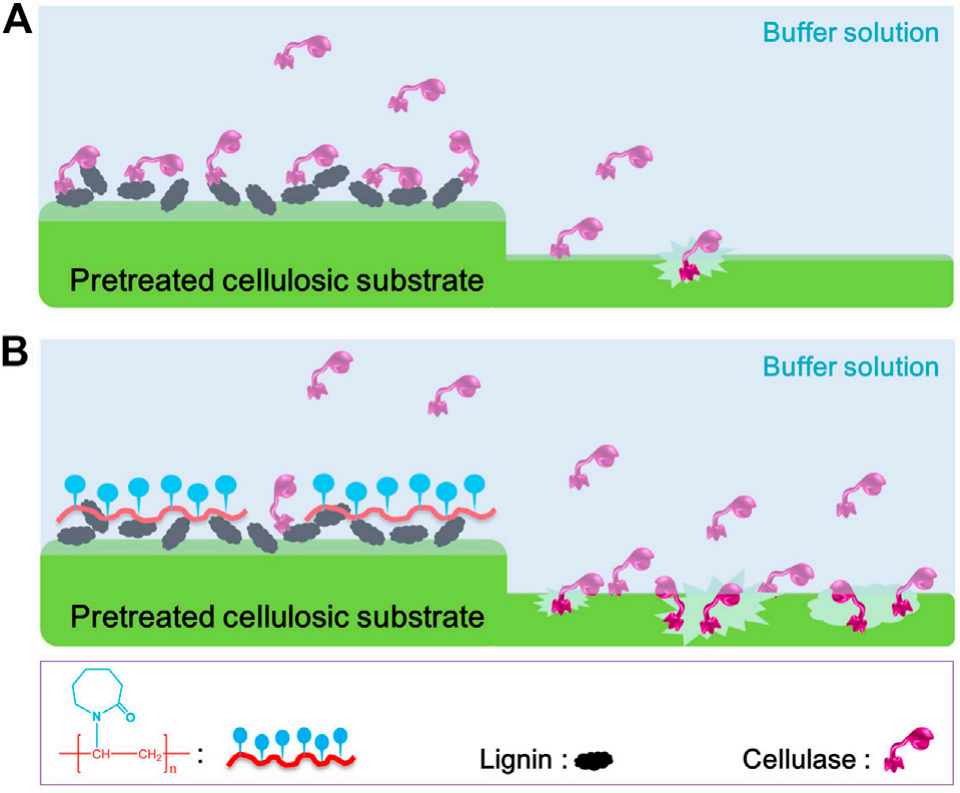
Proposed mechanisms for **(A)** the inhibition of PSA lignin and **(B)** promotion of PNVCL to the enzymatic hydrolysis efficiencies of PSA-Ba substrate.

Based on these results, we speculate that the non-productive adsorption of PSA lignin to cellulase is mainly driven by the hydrophobic interaction, which inevitably decreased the enzymatic hydrolysis efficiency of PSA-Ba substrate (Figure 7A). Moreover, the mechanisms of PNVCL for shielding lignin and inhibiting enzyme adsorption (Figure 7B) can be also reasonably proposed as: (1) the hydrophobic polyolefin chains of PNVCL facilitate efficient blocking of PSA lignin through hydrophobic interactions; (2) PNVCL adsorbed on the surface of lignin can further inhibit the non-productive adsorption of cellulase via electrostatic repulsion and/or hydration; (3) more free enzyme molecules will be available to hydrolyze cellulose, thereby improving its enzymatic hydrolysis efficiency.

## Conclusion

PSA pretreatment can effectively improve the accessibility of bamboo substrate to enzymes through removing non-cellulosic components (hemicellulose and lignin). However, the lignin residues deposited on the surface of the PSA-Ba substrate caused serious non-productive adsorption to enzymes, which significantly reduced enzymatic hydrolysis efficiency. PNVCL, a non-ionic surfactant, was developed as a novel additive for enzymatic hydrolysis. Similar to intensively-studied tween 20 and BSA additives, the addition of PNVCL during enzymatic hydrolysis of cellulose could substantially reduce cellulase loadings as compared to that without additive for achieving remarkable cellulose EC. In general, PNVCL could effectively block PSA lignin and prevent non-productive adsorption of enzymes through intermolecular non-covalent interactions, which would reduce the loading of enzymes and therefore facilitate the economics of PSA pretreatment-based biorefinery.

## Data Availability

The original contributions presented in the study are included in the article/supplementary material, further inquiries can be directed to the corresponding authors.
